# Electrophysiological evidence for the effects of pain on the different stages of reward evaluation under a purchasing situation

**DOI:** 10.3389/fpsyg.2022.943699

**Published:** 2022-09-27

**Authors:** Qingguo Ma, Wenhao Mao, Linfeng Hu

**Affiliations:** ^1^School of Management, Zhejiang University of Technology, Hangzhou, China; ^2^Institute of Neural Management Sciences, Zhejiang University of Technology, Hangzhou, China; ^3^School of Management, Zhejiang University, Hangzhou, China

**Keywords:** pain, reward, neuromarketing, event-related potentials, FRN, P300

## Abstract

Pain and reward have crucial roles in determining human behaviors. It is still unclear how pain influences different stages of reward processing. This study aimed to assess the physical pain’s impact on reward processing with event-related potential (ERP) method. In the present study, a flash sale game (reward-seeking task) was carried out, in which the participants were instructed to press a button as soon as possible to obtain the earphone (a reward) after experiencing either electric shock or not and finally evaluated the outcome of their response. High-temporal-resolution electroencephalogram data were simultaneously recorded to reveal the neural mechanism underlying the pain effect. The ERP analyses revealed that pain affected the feedback processing reflected by feedback-related negativity (FRN) and P300. Specifically, participants in the nopain situation exhibited greater FRN discrepancy between success and failure feedbacks relative to that in the pain situation. Moreover, the P300 amplitude was enhanced in the nopain condition compared to the pain condition regardless of the feedback valence. These results demonstrate that the pain reduced the sensitivity to the reward valence at the early stage and weakened the motivational salience at the late stage. Altogether, this study extends the understanding of the effect of pain on reward processing from the temporal perspective under a purchasing situation.

## Introduction

Pain and reward are both powerful motivators for human behaviors. Generally, the stimuli that trigger painful experiences induce the avoidance behavior, whereas stimuli that are associated with the reward induce the approach behavior (Spielberg et al., 2012). Numerous studies have explored the mechanisms underlying the processing of pain and reward parallelly, while recent interests are focused on how pain affects the neural responses of the reward processing (Becker et al., 2017; Wang et al., 2020).

### The debate regarding the effect of pain on reward processing

Specifically, some studies have found pain would enhance the neural activation for reward processing due to the aggregated motivational salience (Lang, 1995; Cui et al., 2016). For example, a recent fMRI study showed that after undergoing the pain, the participants’ neural activity in the medial prefrontal cortex (mPFC) responding to win outcome was increased (Wang et al., 2020). Additionally, an ERP study found that concurrent pain information could modulate the feedback processing in a gambling task, revealing that neural activities reflected by N1 and P300 were enhanced in reward condition compared with loss condition when the feedback was manifested by the pain stimuli(Cui et al., 2016).

On the contrary, several studies have demonstrated that in the situation involving both reward and pain, they compete against each other, thus leading to the inhibiting effect of pain on reward processing (Desimone, 1995; Styles, 1997; Van Damme et al., 2012; Choi et al., 2015). For example, a previous study found that when pain and reward occurred simultaneously, anterior cingulate cortex (ACC; Talmi et al., 2009) and ventral striatum (Gorka et al., 2018; Kim et al., 2020) showed a significant attenuation in sensitivity to monetary reward. Besides, the Choi et al. (2013) fMRI study found that the interaction between reward and pain processing activated several brain regions, such as anterior insula, dorsal ACC, and the activities of these regions during the reward processing were attenuated under the higher level of pain (Choi et al., 2013). In a recent fMRI study, researchers found pain would lead to the dysfunction of the mesolimbic structure (e.g., striatum), which is involved in encoding motivational salience. In detail, participants who experienced pain exhibited dampened striatum activation during reward and loss trials of the Monetary Incentive Delay (MID) task (Kim et al., 2020). These results indicate that the pain weakens the reward processing, tracked by reduced activities of brain regions related to the reward processing. In addition to the enhancing and inhibiting impacts of the pain on reward processing that have been discussed in some studies, there is also empirical evidence supporting the null effect of the pain on reward processing (Kim and Anderson, 2020). To sum up, the findings of how pain influences reward processing are not consistent across different studies, and this question still needs further exploration.

### Reward evaluation under purchasing situations

The consumption process is also a self-reward process. Such kind of reward can be a hedonic reward (e.g., massage) or a utilitarian reward (e.g., grocery purchases) (Mukhopadhyay and Johar, 2009). A previous study indicated that approximately 20% of consumers suffer from pain at any given time (Goldberg and McGee, 2011). However, few studies focus on the mechanism of the pain’s effect on consumer’s behavior. The existing studies have also not reached an agreement on how pain will affect consumption decisions. For example, previous researches indicate that physical pain can increase consumers’ purchase consumption intention (Darbor et al., 2016; Chan, 2021). While another research suggests physical pain can compete against reward processing during purchase, the presentation of a loving brand’s logo can help participants decrease pain perception (Reimann et al., 2017).

### Spatiotemporally distinct value systems

Recent studies have found that reward feedback processing can be divided into different stages. For example, a study found two spatiotemporally distinct value systems in the brain underlying the reward feedback processing (Fouragnan et al., 2015). Specifically, an early system was activated only by the negative outcomes, and a later system differentially suppressed or activated the regions of the reward network in response to negative and positive outcomes. Furthermore, another electroencephalogram (EEG)-fMRI study discovered the vmPFC uniquely contributed to a sustained activation profile shortly after outcome presentation, whereas the dmPFC contributed to a later and more peaked activation pattern (Hauser et al., 2015). It is noteworthy that there may be a possibility that the pain plays different roles at different stages of reward processing. However, most previous studies mainly applied the fMRI method to uncover the effect of pain on reward processing based on the activation patterns of the brain related to the reward, but few studies investigate the effect of pain from a temporal perspective. The event-related potentials (ERPs) method possesses the advantage of high temporal resolution compared to the fMRI and has been adopted in many studies about the reward. According to previous ERP studies concerning reward feedback processing, the neural signals of feedback-related negativity (FRN) and P300 (Glazer et al., 2018) have been extensively discussed and can reflect the distinct stages of the reward processing. Therefore, in the current study, we mainly focused on these two signals to reveal the neural mechanism underlying the pain effect on reward processing under purchasing situations.

### ERP components related to reward feedback processing

The FRN is a fronto-central ERP component elicited within 300 ms following the reward feedback onset that has been verified localized to the ACC (Gehring and Willoughby, 2002; Miltner et al., 2003; Luft, 2014; Jin et al., 2020). FRN reflects the early processing of the feedback and is sensitive to the valence of the feedback. Generally, the FRN amplitude is thought to signal greater negativity when reward feedback has gone worse (vs. better) than expected (Yan and Zhou, 2009; Glazer et al., 2018). According to the previous study, enhanced FRN amplitude after negative feedback may index error-provoked attentional control (Krause et al., 2020). Besides, the discrepancy of the FRN amplitude between gain and loss (named as d-FRN) is also used to index the reward processing, and the larger d-FRN can reflect the increased attentional resources devoted to the reward processing (Krause et al., 2020). Due to the limited processing capacity of neural systems, our brains have evolved efficient selection mechanisms that can bias the processing of salient stimuli (Bourgeois et al., 2017). The early processing of the reward could be modulated by the factors associated with attention allocation (Jiang et al., 2021). For example, a previous study found that the positive mood could build additional attention resources and thus alter brain mechanisms of reward prediction errors during performance monitoring, which was tracked by the enhanced d-FRN in the positive mood condition (Paul and Pourtois, 2017). A recent study also found participants exhibited smaller d-FRN in response to the reward feedback after they experienced the pain, and the author attributed this finding to the reduced sensitivity to outcome valence in the pain condition (Jiang et al., 2021).

Another ERP component implicated in reward feedback processing that directly follows the FRN is the P300, a centro-parietal positive-going deflection peaking from 300 to 600 ms (Glazer et al., 2018). Generally, P300 is considered to be associated with attention allocation, and larger P300 amplitude is elicited when more attentional resources activate (Halgren et al., 1995; Polich, 2007). In the reward-related studies, the larger P300 has been reported to reflect the increased attention to motivationally salient stimuli, such as the monetary gain (Pfabigan et al., 2014). Besides, the P300 is also sensitive to the reward magnitude (Sato et al., 2005; Toyomaki and Murohashi, 2005; Kamarajan et al., 2009). For example, a previous study found that participants exhibited larger P300 after winning a larger magnitude money compared to smaller ones (Sato et al., 2005).

Taken together, FRN can be associated with an early automatic process, and P300 is supposed to be related to the later cognitive processes in response to the reward feedback. According to previous studies, the pain could enhance the reward processing by aggregating the motivational salience (Gandhi et al., 2013; Wang et al., 2020), or could inhibit the reward processing by competing for the attention resource (Gong et al., 2020). Since both early and later processing of the reward can be influenced by the attention resource and the later processing is more sensitive to the motivational salience of the reward (Sato et al., 2005; Cavanagh, 2015). This current study assumed that the effects of pain might manifest differently at early and later stages of the reward processing, resulting in the dynamic patterns of the FRN and P300. To test this hypothesis, the current study adopted an ERPs experiment to explore the temporal substrates of reward evaluation after undergoing different levels of physical pain. Specifically, a flash sale game (a variant of the reward-seeking task) was designed to evoke reward processing, in which the participants were instructed to press the button as soon as possible to obtain an earphone (a reward) after experiencing either electric shock or not and finally evaluated the feedback of their response, consisting of success and failure. The FRN and P300 in response to the different valences of feedback under the nopain and pain conditions were analyzed.

## Materials and methods

### Participants

Thirty-eight right-handed students (16 male) aged 18–26 years old (*M* = 21.657, *SD* = 2.290) participated in this experiment. They had normal or corrected-to-normal vision and did not have any history of neurological disorders or mental diseases. This study was approved by the Internal Review Board of the Institute of Neuromanagement in Zhejiang University of Technology. Informed consents were obtained from all participants before the experiment formally started. Data from two participants were discarded because of excessive recording artifacts, resulting in thirty-six valid participants for the final data analysis.

### Experiment procedure

In order to investigate the impact of pain on reward processing, a flash sale game (a variant of the reward-seeking task) was implemented in this study, which offered the success or failure outcome according to participants’ responses. Specifically, participants were instructed to press the button to get an earphone. They were announced that the faster they respond, the higher chance they can get the earphone, i.e., a reward. Eighty pictures of the earphone were collected online. To avoid the influence of earphone pictures on participants’ motivation, earphones’ information (brand, model, size, etc.) was removed from the picture. And all pictures have a white background and were processed into the same size. For the procedure of pain induction, one identical electrical stimulation device (mode: YRKJ-F1002; Yiruikeji Co. Ltd., Zhuhai, China) was used to exert shocks with two electrodes attached to the participant’s left index finger in the pain trials. Before the experiment, the participants were provided with written experimental instructions. After they read the instructions, the experimenter explained the task and conducted a shock calibration procedure to determine each participant’s electric shock intensity that was “feeling mildly painful” as previous studies did (Neddermeyer et al., 2008; Schmidt et al., 2016). Specifically, participants received increasing electrical shock starting from 1 mA with increments of 1 mA and with the time interval of about 5 s until participants reported verbally that they felt mildly painful. Finally, the 70% of each participant’s highest allowable shock intensity was used in the formal experiment (*M* = 13.778 ± 3.833 mA).

**Figure 1:**
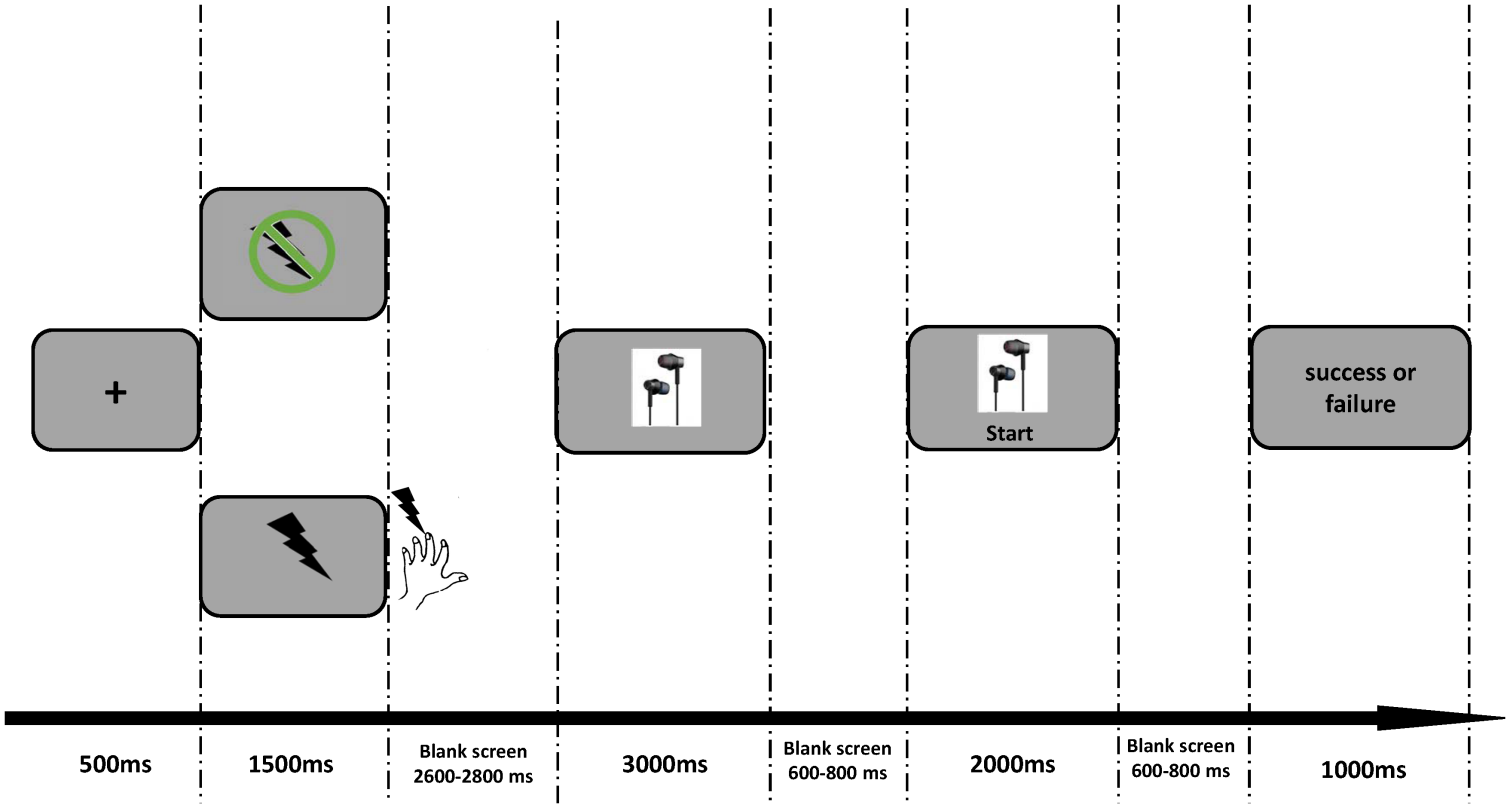
Experimental design. For each trial, the pain or nopain cue was firstly presented for 1,500 ms. Then the electrical shock (0.1 Hz) was conducted only after the pain cue disappeared. Following a 2,600–2,800 ms blank, the earphone picture appeared on the screen for at least 3,000 ms. The participants were told to press the number “2” on the keyboard as soon as the “start” button appeared. Finally, the system presented the feedback of success or failure for 1,000 ms.

As illustrated in Figure 1, each trial began with a fixation appearing for 500 ms on the silver screen, followed by the 1.5 s cue indicating electrical shock or not. In a pain condition, the participants would receive an electrical pulse (0.1 Hz) as soon as the electrical cue (lightning icon shown in Figure 1) disappeared. This electrical pulse lasted for 100 ms. In the nopain condition, participants would not receive stimulation after the no-electrical cue (lightning icon with prohibition sign). Following the cue, a blank screen was presented for 2.6 ~ 2.8 s. Then a picture of earphones was shown for 3 s. The participants were instructed to press the ‘2’ button in 2 s only when a ‘start’ appeared below the earphone picture and were told that the faster they responded, the higher chance they could get the earphone. Actually, the outcome in each trial was arranged pseudo-randomly by the program as long as the participants pressed the button within 2 s, showing the words “success” or “fail” for 1 s. A trial would be deemed as a failure if the participants did not make a response within the 2 s time limit and these trials were deleted for further analysis. The experiment consisted of 4 blocks, each containing 40 trials. After the experiment, one trial was randomly selected from 160 trials. If the selected trial was successful, the participants would get 50 yuan; otherwise, they would get 40 yuan.

### EEG recording

EEG data were continually collected during the task by using 64 Ag-AgCl scalp electrodes placed according to the International 10–20 system (Neuroscan Synamp2 Amplifier; bandpass filter: 0.01–250 Hz; sampling rate: 500 Hz). The electrode-to-skin impedances were kept below 10 kΩ for all electrodes. Electro-oculographic signals were simultaneously recorded using surface electrodes to monitor ocular movements and eye blinks.

### Data analysis

EEG data were preprocessed by using the MNE, a Python-based open-source toolbox for EEG data analysis (Gramfort et al., 2013). Firstly, continuous EEG data were bandpass filtered between 1 and 30 Hz (Ma et al., 2018). After the bandpass filter, EEG trials were re-referenced off-line to the average of the left and the right mastoids. Then, EEG epochs that were time-locked to the onset of the feedback of response were extracted into a 1,000 ms time window (200 ms prestimulus and 800 ms poststimulus), and baseline corrected using the prestimulus interval. EEG epochs were visually inspected, and trials contaminated by eye blinks and movements were corrected using an independent component analysis (FastICA) algorithm (Hyvarinen, 1999).

Single-trial ERP waveforms elicited in four conditions, including pain-success, pain-failure, nopain-success, nopain-failure were averaged separably for each participant. Subsequently, single-participant averaged ERP waveforms were averaged to obtain group-level waveforms, and scalp topographies were computed by spline interpolation. Dominant ERP components, including FRN and P300, were identified according to the scalp topographies of grand average ERP activity and previous studies (Glazer et al., 2018). We analyzed the amplitude of FRN with the pooled electrodes including F1, FZ, F2, FC1, FCZ and FC2. In detail, a 2 × 2 within-participant repeated-measures analysis of variance (ANOVA) on the mean amplitude of FRN (time window: 160–210 ms) was conducted with feedback (success vs. failure) and pain (pain vs. nopain) as within-participant factors. Similar ANOVA was conducted on the mean amplitude of P300 during 350–450 ms by pooling electrodes including C1, CZ, C2, CP1, CPZ and CP2 with feedback (success vs. failure) and pain (pain vs. nopain).

## Results

### The results of FRN

**Figure 2:**
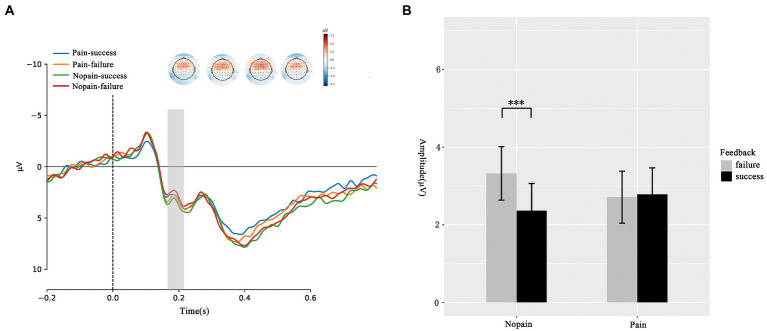
FRN responses during the feedback screen. **(A)** Average ERP waveforms and scalp topographies for pain (pain vs. nopain) and feedback (success vs. failure) during the task. Displayed waveforms were measured at FCZ. Amplitudes of dominant component FRN elicited by the feedback were compared. **(B)** Histogram for the FRN in the four conditions. ^***^p < 0.01.

We first compare the amplitude of FRN under four conditions. As illustrated in Figure 2, the ANOVA of FRN suggested that the main effects of feedback (*F*_(1, 35)_ = 2.436, *p* = 0.128, ƞ^2^_p_ = 0.065) and pain (*F*_(1, 35)_ = 0.106, *p* = 0.747, ƞ^2^_p_ = 0.003) were not significant. The interaction effect between feedback and pain was significant (*F*_(1, 35)_ = 27.68, *p* = 0.047, ƞ^2^_p_ = 0.108). The simple effect analysis showed that in nopain condition, failure feedback elicited greater FRN amplitude in contrast to success feedback (failure: 2.361 ± 0.703 μV vs. success: 3.323 ± 0.691 μV, *p* = 0.009). However, this difference was not significant in pain condition (failure: 2.784 ± 0.684 μV vs. success: 2.712 ± 0.669 μV, *p* = 0.861). Which indicated pain diminish the valence induced amplitude difference. To clearly show the difference of feedback effect between pain and nopain conditions, we further analyzed the d-FRN (FRN_success_ – FRN_failure_) and found the nopain condition evoked an obviously larger d-FRN compared with the pain condition (pain: −0.072 ± 0.408 μV vs. nopain: 0.961 ± 0.348 μV, *p* = 0.047).

### The results of P300

**Figure 3:**
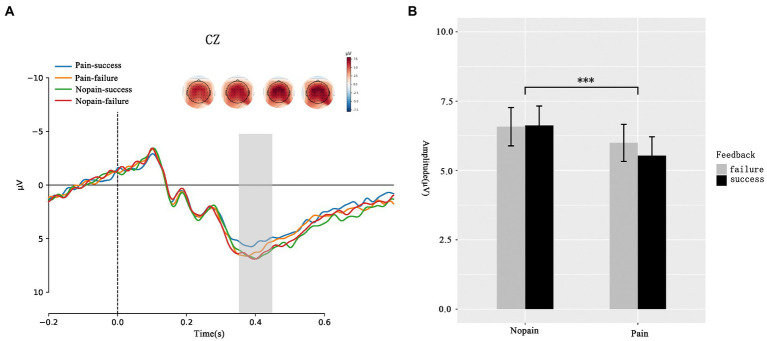
P300 during the feedback screen. **(A)** Average ERP waveforms and scalp topographies for pain (pain vs. nopain) and feedback (success vs. failure) during the task. Displayed waveforms were measured at CZ. Amplitudes of dominant component P300 elicited by the feedback were compared. **(B)** Histogram for the P300 in the pain and nopain conditions. ^***^p < 0.01.

Beyond FRN, we analysis the amplitude of P300. As presented in Figure 3, the ANOVA analysis of P300 showed that the main effect of feedback (*F*_(1, 35)_ = 0.273, *p* = 0.604, ƞ^2^_p_ = 0.08) was not significant while the main effect of pain (*F*_(1, 35)_ = 8.741 *p* = 0.008, ƞ^2^_p_ = 0.183) was significant. Participants exhibit larger P300 amplitude in nopain condition than in pain condition (pain: 5.761 ± 0.982 μV vs. nopain: 6.600 ± 0.962 μV). No interaction effect between feedback and pain was found (*F*_(1, 35)_ = 1.023, *p* = 0.319, ƞ^2^_p_ = 0.028).

## Discussion

This study intended to investigate the impact of physical pain on the different stages of reward processing under a purchasing situation with the high-temporal-resolution EEG data. The ERP results demonstrated that the pain indeed influenced the brain activities underlying the reward processing. Specifically, larger FRN difference between success and failure feedbacks were observed in nopain condition than that in pain condition. Besides, only the pain effect on P300 response was found with a significantly more positive deflection in nopain than pain condition. These results indicated that the pain can mitigate reward processing. Specifically, at the early stage, pain interferes with the detection of reward feedback valence, while at the late stage, the pain reduces the motivation salience of reward feedback. To our knowledge, this study firstly provided evidence of neural activities from the temporal perspective to deepen our understanding of how pain influences reward processing under a purchasing situation.

At the early reward feedback processing stage, we observed a larger FRN discrepancy between success and failure feedbacks in nopain condition, which was in accordance with previous findings that the FRN is indicative of the early automatic recognition of feedback valence and exhibited more negativity when the feedback has gone worse than expected (Luft, 2014; Glazer et al., 2018). Therefore, more negative FRN amplitude evoked by the failure feedback compared to that by the success feedback under nopain condition indicated the participants could distinguish different outcomes early (Hajcak et al., 2006). However, we did not observe such an effect of feedback valence under the pain condition. Besides, we also did not find the pain effect on the FRN. These results might be explained by the impact of the pain on the attention system, which then modulated the reward feedback processing. Previous studies have found that attention is quite vulnerable to pain, as the pain would interrupt the cognitive processing and capture the attention that might be allocated elsewhere (Blöchl et al., 2015). In this study, the participants experienced the electric shock ahead, and this physical state of pain could last for a while (Gong et al., 2020), which still occupied the attention at the early stage of reward feedback processing and resulted in the diminished sensitivity to the feedback valence. Furthermore, a previous study found negative stimuli had the deleterious impact on the processing of reward, which was attributed to the rapid attentional competition between reward and negative stimuli (Yokoyama et al., 2015). Therefore, the diminished d-FRN in our study may reflect a similar mechanism: pain affects the processing of reward by occupying individual attention resources. This result is also consistent with the previous study’s prediction that changes in attention resources can modulate individuals’ performance monitoring and reward prediction errors, reflected by the manifestation of FRN (Paul and Pourtois, 2017). To sum up, the smaller d-FRN observed after the pain experience may be attributed to the limited cognitive recourse under the pain condition and indicated the impaired ability to detect the valence of reward feedback at the early stage.

In the current study, we found a larger P300 amplitude in the nopain condition than in the pain condition, which provided evidence regarding the impaired effect of pain on the attention resource. Our FRN results indicated that the pain took up attention resources at the early stage of reward processing. This influence might last at the later stage, which was mirrored by the significant pain effect on P300. A previous study also found pain could cause a significant reduction of oddball-evoked P300 (Rosenfeld and Kim, 1991). P300 is an index of attention allocation, and the enlarged P300 reveals more attention allocated to the current stimuli processing (Polich, 2007). Therefore, the less positive P300 amplitude in the pain condition than that in the nopain condition implied that participants devoted fewer resources to process the reward feedback under the pain condition. Our study did not observe the feedback valence on P300 both in pain and nopian conditions. We considered the following possible explanation. According to the independent coding hypothesis, the valence and magnitude of the feedback are coded independently (Yeung and Sanfey, 2004). P300 amplitude is larger after presenting motivationally salient stimuli (Groot and Strien, 2019) and more sensitive to reward magnitude or probability (Yeung and Sanfey, 2004; Hajcak et al., 2005). For example, Pfabigan et al. (2014) found that higher motivational stimuli, regardless of gain or loss conditions, can capture more attention and elicit a more positive P300 (Pfabigan et al., 2014). Besides, Sato et al. (2005) found that the amplitude of P300 increased with the magnitude of reward or penalty but was unaffected by the valence of outcome in a MID task (Sato et al., 2005). Another study discovered that P300 was larger when feedback was perceived as infrequent, while P300 did not differ for positive and negative feedback (Hajcak et al., 2005). According to previous studies, the motivational salience of feedback is sensitive to magnitude (Sato et al., 2005; Toyomaki and Murohashi, 2005; Kamarajan et al., 2009) and probability (Yeung and Sanfey, 2004; Hajcak et al., 2005). It demonstrates that P300 may be more related to motivational salience rather than valence (Pfabigan et al., 2014). In our experiment, the feedbacks of success and fail had the same magnitude and probability of appearing. Therefore, these successes and fail feedbacks might possess similar motivational salience, resulting in valence’s null effect on the P300. Besides, smaller P300 in pain conditions also could indicate that the pain reduced the motivational salience of the reward feedback, which was consistent with previous study’s finding that pain decreased the striatum activity that was involved in encoding motivational salience for the reward (Kim et al., 2020). In conclusion, pain impairs reward processing at the later stage.

Altogether, the results of FRN and P300 indicated that the pain could inhibit the reward processing under a purchasing situation by distracting the attention, which was consistent with the competitive explanation of the interaction between pain and reward proposed in some studies (Desimone, 1995; Styles, 1997; Van Damme et al., 2012; Choi et al., 2015). However, this impaired effect manifests differently in the early and later stages. At the early stage, pain mitigates the rapid recognition of reward valence reflected by smaller d-FRN while reduces motivational salience at the later stage reflected by smaller P300. These results were in line with prior EEG-fMRI studies that suggested two temporally distinct neuronal components associated with reward processing, which might react differently to non-reward related factors (Gentsch et al., 2009; Doñamayor et al., 2011; Fouragnan et al., 2015).

However, some limitations exist in this study. A recent study found EEG can reflect complex and dynamic pain-related stimulus processes at the reward feedback stage (Hoy et al., 2021). The method used in the current study is relatively simple and lacks a dynamic system association analysis at the individual level. An in-depth analysis of the dynamic feature of the EEG signal in future studies may help researchers get more interesting conclusions. Meanwhile, the participants in this study received acute pain in the experiment. In future research, a similar paradigm could be used to investigate the effects of chronic pain on reward evaluation under a purchasing situation to obtain more generalized conclusions.

## Conclusion

Taken together, this study integrated the high-temporal-resolution ERP method into the investigation of how pain influenced reward processing by applying a flash sale game. The ERP results indicated that the reward processing under the purchasing situation was deeply impacted by pain. Specifically, pain regulates reward processing by influencing the individuals’ attention resource, thus reducing the sensitivity to the reward valence at the early stage and weakening motivational salience at the later stage. As current results indicate, pain can influence the purchase motivation of consumers. Therefore, the corporate marketing staffs need to consider the potential impact of pain on the implicit psychological process of consumers when formulating marketing strategies. Consider incorporating pain-related factors into the user behavior prediction based on big data.

## Data availability statement

The raw data supporting the conclusions of this article will be made available by the authors, without undue reservation.

## Ethics statement

The studies involving human participants were reviewed and approved by the Internal Review Board of the Institute of Neuromanagement in Zhejiang University of Technology. The patients/participants provided their written informed consent to participate in this study.

## Author contributions

LH and QM designed the experiments, wrote the manuscript, and contributed materials and analysis tools. WM performed the experiments, analyzed the data, and drew figure. All authors contributed to the article and approved the submitted version.

## Funding

This study was supported by the National Natural Science Foundation of China (no. 71942004 and 72002202), the Humanities and Social Sciences Foundation of the Ministry of Education of China (no. 20YJC630040), the Economic and Technological Research Institute of State Grid Zhejiang Electric Power Company, Ltd. (no. SR1320190057), and the project of science and technology innovation of Zhejiang province (no. 2021R403051).

## Conflict of interest

The authors declare that the research was conducted in the absence of any commercial or financial relationships that could be construed as a potential conflict of interest.

## Publisher’s note

All claims expressed in this article are solely those of the authors and do not necessarily represent those of their affiliated organizations, or those of the publisher, the editors and the reviewers. Any product that may be evaluated in this article, or claim that may be made by its manufacturer, is not guaranteed or endorsed by the publisher.
